# Biology, Ecology, and Behavior of Rusty Grain Beetle (*Cryptolestes ferrugineus* (Stephens))

**DOI:** 10.3390/insects14070590

**Published:** 2023-06-30

**Authors:** Vimala S. K. Bharathi, Fuji Jian, Digvir S. Jayas

**Affiliations:** Department of Biosystems Engineering, University of Manitoba, Winnipeg, MB R3T 5V6, Canada; vimala.bharathi@outlook.com (V.S.K.B.); fuji.jian@umanitoba.ca (F.J.)

**Keywords:** *Cryptolestes ferrugineus*, rusty grain beetle, stored grain pest, biology, ecology

## Abstract

**Simple Summary:**

The rusty grain beetle, *Cryptolestes ferrugineus* (Stephens), is a common pest found worldwide that can adapt to various climates. This insect poses a significant economic threat, making it crucial to understand its biology, ecology, and behavior to develop effective management strategies. To gain insights, a comprehensive review of the existing literature about *C. ferrugineus* was performed utilizing databases such as Web of Science and Scopus. The review covered publications from 1949 to 2023 and highlighted the global importance of *C. ferrugineus* through its presence in over 110 countries. The article provides a comprehensive examination of the insect’s biology and ecology, highlighting influential factors. A summary of the research performed on the interspecific interaction of *C. ferrugineus* with other organisms has also been presented. Mathematical models focusing on population dynamics and movement behavior are also presented. Finally, the article outlines the potential directions for future work, aiming to deepen our understanding of *C. ferrugineus* and aid in the development of improved management strategies.

**Abstract:**

*Cryptolestes ferrugineus*, the rusty grain beetle, is a cosmopolitan pest that has adapted to cool and warm climates due to its unique biology, ecology, and behavior. The rusty grain beetle is a pest of high economic importance; hence, understanding their biology, ecology, and behavior could be useful in designing effective management strategies. An extensive literature survey was conducted using the databases Web of Science and Scopus. Information on country-wise publications from 1949 to 2023 on *C. ferrugineus* was provided, and a table illustrating the distribution of *C. ferrugineus* was also presented to demonstrate the global significance of *C. ferrugineus.* We overviewed their life stages, morphology, and factors influencing their biology, ecology, and behavior, such as refuge-seeking behavior, flight activity, mating behavior, interspecific interaction with other species, movement, and distribution. Mathematical models focusing on *C. ferrugineus* population dynamics and movement were also presented. In order to advance our knowledge on *C. ferrugineus*, the following possible avenues for future research were outlined: application of molecular markers and population genetic approaches to understand their evolutionary history; mechanisms responsible for adaptation and resistance to insecticide; interspecific interaction in storage facilities and wider landscapes; and identification of microbial roles in the ecology, behavior, and control of *C. ferrugineus*.

## 1. Introduction

The rusty grain beetle, *Cryptolestes ferrugineus* (Stephens) (Coleoptera: Laemophloeidae), is considered a significant threat to the global food supply chain, causing significant economic losses and food waste. The insect is known for its reddish-brown coloration, adaptability to wide environmental conditions, cosmopolitan nature, unique behavior, and reproductive capabilities. Despite its importance, a comprehensive publication on the biology, ecology, and behavior of *C. ferrugineus* is currently lacking in the literature. In 1949, Rilett [1] summarized the biology of *C. ferrugineus* in detail. In 2009, Jian and Jayas [2] provided a detailed review focusing mainly on the movement of the insect. Several aspects of its behavior have been explored by various researchers around the world. Hence, the current review aims to summarize the relevant literature on the biology and ecology of *C. ferrugineus* to provide a detailed comprehension of one of the most important pests in the world. The information presented in this review will be useful for researchers, pest management professionals, and policymakers to develop effective and sustainable strategies to control the pest and reduce economic losses and food waste.

## 2. Literature Survey and Geographical Distribution

**Figure 1:**
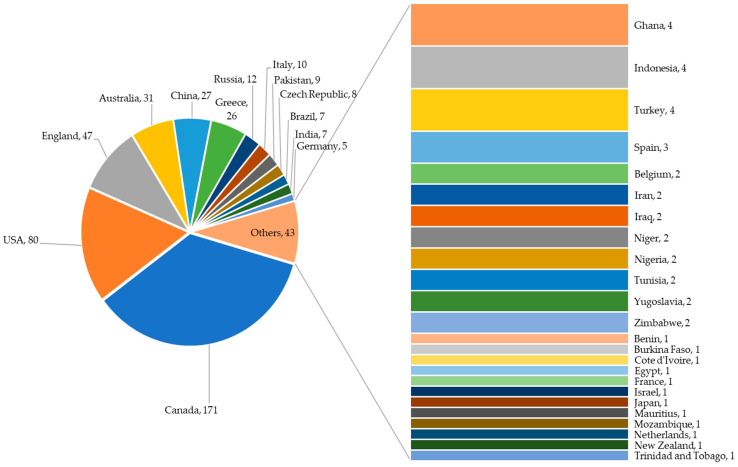
The number of publications on *Cryptolestes ferrugineus* from different countries, compiled using the data retrieved from 483 publications from Web of Science and Scopus from 1949 to 2023 (as of 1 January 2023). The countries were listed based on the affiliation of the first author.

**Figure 2:**
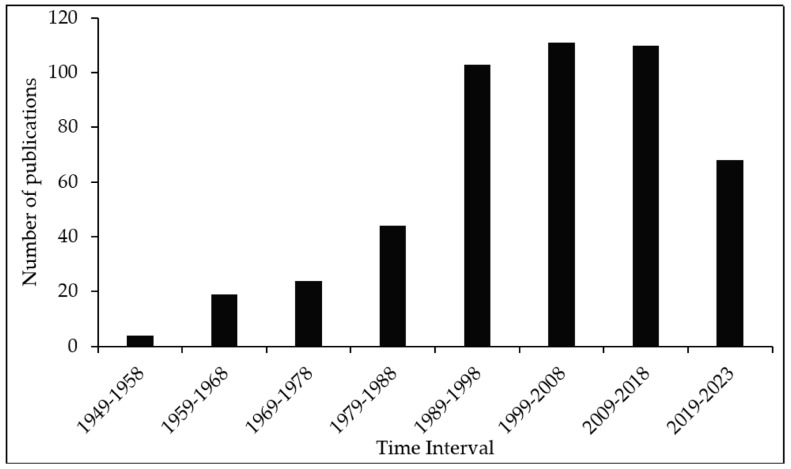
The number of publications on *Cryptolestes ferrugineus* at different time intervals, compiled using the data retrieved from 483 publications from Web of Science and Scopus from 1949 to 2023 (as of 1 January 2023).

The review of literature was conducted using the databases Web of Science and Scopus with search terms such as “Rusty grain beetle”, “*Cryptolestes ferrugineus*” and “*Laemophloeus ferrugineus*”. On 1 January 2023, a total of 380 and 412 articles (in English and other languages, such as Chinese, Czech, French, German, Portuguese, Russian, and Turkish) were retrieved from the Web of Science and Scopus databases, respectively. The articles from both sources were merged, and the duplicates were removed. A total of 483 distinct research publications from 1949 to 2023 were compiled. To categorize the country of investigation, the affiliation of the authors was taken into consideration. For an article with authors from multiple countries, the country of the first author was assumed. From Figure 1, it can be observed that most of the literature on *C. ferrugineus* was published in Canada (171), followed by the U.S. (80) and the UK (47). The surge in the number of publications on *C. ferrugineus* over the years (Figure 2) highlights the economic importance of this insect. Even though a literature search in well-known and reputable databases like Web of Science and Scopus is a valid approach for conducting a systematic review, restricting a literature search to Web of Science and Scopus may introduce some bias. The additional literature that is not included in Web of Science and Scopus has been retrieved from Google Scholar and cited appropriately in the current article.

The rusty grain beetle has been reported in more than 110 countries (Table 1) and can be found in almost any country of the world, ranging from humid to dry as well as cool to warm climates, due to its ability to develop in wide environmental conditions and the world trade. The climatic plasticity index of *C. ferrugineus* is 570 [3], indicating its remarkable ability to adapt to changes in environmental conditions. Among the 195 countries listed in FAO [4], we could not find the sources to confirm the presence of *C. ferrugineus* in 82 countries on different continents, such as Africa (19), Asia (18), Europe (12), North America (12), South America (5), and Oceania (15). Considering the nature of *C. ferrugineus*, it could possibly be established in most of those countries as well. In countries like Canada with cold winters, the establishment of other stored grain pests was limited. However, *C. ferrugineus* has been identified as one of the major grain pests in western Canada since the early 1940s. The species has been found in Roman archaeological excavations in England and Israel [5]. Since it is widely distributed in the world, researchers have extensively explored its ecology, behavior, and control techniques.

Even though *C. ferrugineus* can develop on botanicals such as *Pimpinella anisum* (L.) (anise), *Hibiscus sabdariffa* (L.) (roselle), *Coriandrum sativum* (L.) (coriander), *Matricaria chamomilla* (L.) (chamomile), *Glossostemon bruguieri* (Desf.) (mogat), and *Origanum majorana* (L.) (marjoram), it thrives in stored grain [6]. *Cryptolestes ferrugineus* mainly infests the following stored products: wheat, maize, barley, sorghum, oats, flour, groundnuts, beans, cassava, rice, sunflower seeds, palm kernels, cacao beans, and cotton seeds. They are found in farms, maltings, mills, warehouses, storage bins, and other storage structures [7]. At their optimum temperature (33 °C) [3], *Cryptolestes ferrugineus* can rapidly multiply and damage the grain, leaving the hollow grain kernels as leftovers.

**Table 1:** Countries where *Cryptolestes ferrugineus* has been recorded.

Countries	References
Afghanistan	[8,9]
Algeria	[7,9]
Angola	[10]
Argentina	[7,9]
Armenia	[9]
Australia	[7,11,12]
Austria	[9,13]
Azerbaijan	[14]
Bangladesh	[9,15,16]
Belarus	[17]
Belgium	[9,18,19]
Belize	[7]
Benin	[20,21]
Botswana	[22]
Brazil	[7,23]
Bulgaria	[24]
Burkina Faso	[25,26]
Cabo Verde	[27]
Cameroon	[28]
Canada	[7,9,29,30,31]
Chad	[32]
Chile	[9]
China	[7,33]
Colombia	[34,35]
Congo	[9]
Costa Rica	[36]
Cote d’Ivoire	[28,37]
Croatia	[38]
Cuba	[39]
Czech Republic	[9,40,41]
Denmark	[9,42]
Dominican Republic	[28]
Ecuador	[9,15]
Egypt	[6]
El Salvador	[43]
Estonia	[44]
Ethiopia	[9,15,45]
Finland	[9,44]
France	[46]
Gambia	[7]
Germany	[9,47]
Ghana	[9,26,48,49]
Greece	[9,50,51]
Guinea	[52]
Guyana	[7,9]
Hungary	[53]
Iceland	[54] recited from [55]
India	[9,56]
Indonesia	[57]
Iraq	[58] recited from [59]
Iran	[9,60]
Ireland	[61]
Israel	[9]
Italy	[62]
Jamaica	[7]
Japan	[9,63,64]
Jordan	[65]
Kazakhstan	[66]
Kenya	[7,9,15,67]
Lithuania	[9,68]
Luxembourg	[44]
Madagascar	[9]
Malawi	[7,9]
Malaysia	[7,9]
Mali	[9,12]
Malta	[69]
Mexico	[9,70,71]
Montenegro	[44]
Morocco	[7,9]
Mozambique	[72]
Myanmar	[7]
Namibia	[73]
Nepal	[74]
Netherlands	[9,75]
New Zealand	[7,76]
Nicaragua	[9,15,77]
Niger	[21]
Nigeria	[9,78,79]
Norway	[9,44]
Pakistan	[9,80]
Peru	[7,9,15]
Philippines	[9,15]
Poland	[9,81]
Portugal	[7,9,44]
Republic of Korea	[82]
Republic of Moldova	[83]
Russia	[7,9,84]
Saudi Arabia	[9,15,45]
Senegal	[85]
Sierra Leone	[9,15]
Singapore	[7,9,45]
Slovakia	[9]
Somalia	[9,86]
South Africa	[7,9]
Spain	[9,87,88]
Sri Lanka	[7,9,89]
Sudan	[7,9,12,45]
Sweden	[9,90]
Switzerland	[9,44]
Tanzania	[7,9]
Thailand	[7]
Timor-Leste	[91]
Togo	[26]
Tunisia	[7,9]
Turkey	[92,93,94]
Uganda	[9]
Ukraine	[68,95]
United Arab Emirates	[9]
United Kingdom	[96,97]
United States of America	[98,99,100,101]
Uruguay	[7,9]
Vietnam	[9,102]
Yemen	[9,15]
Zambia	[7]
Zimbabwe	[7,9,103]

## 3. Taxonomic Hierarchy, Identification, and Synonyms

The rusty grain beetle, also known as the rust-red grain beetle or flat grain beetle, was initially described by James Francis Stephens in 1831 under the name *Cucujus ferrugineus*. *Cryptolestes* was listed as a subgenus under the genus *Laemophloeus,* and the insect was referred to as *Laemophloeus ferrugineus* (Stephens) by Leng, whereas Casey claimed that *Cryptolestes* could be referred to as an individual genus due to its distinct nature, which was agreed upon by other researchers such as Sheppard [1]. Available synonyms are *Cucujus monilicornis* (Stephens, 1831), *L. concolor* (Smith, 1851), *L. obsoletus* (Smith, 1851), *L. carinulatus* (Wollaston, 1877), *L. emgei* (Reitter, 1887), and *L. alluaudi* (Grouvelle, 1906) [104]. In the mid-20th century, researchers often used *L. ferrugineus* (Stephens). Currently, *Cryptolestes ferrugineus* (Stephens) is widely used.

The order, suborder, infraorder, superfamily, family, genus, and species of rusty grain beetle are Coleoptera, Polyphaga, Cucujiformia, Cucujoidea, Laemophloeidae, *Cryptolestes,* and *Cryptolestes ferrugineus*, respectively [105]. There are about 50 species in the genus *Cryptolestes* Ganglbauer, 1899, but only nine are considered pests of stored products: *C. capensis* Waltl, 1834; *C. cornutus* Thomas and Zimmerman, 1989; *C. divaricatus* Grouvelle, 1898; *C. ferrugineus*; *C. klapperichi* Lefkovitch, 1962; *C. pusillus* Schönherr, 1817; *C. pusilloides* Steel and Howe, 1952; *C. turcicus* Grouvelle, 1876; and *C. ugandae* Steel and Howe, 1952. Differentiation of *C. ferrugineus* from other *Cryptolestes* spp. could be performed by identifying the morphological differences in those species as listed in Table 2. Different *Cryptolestes* species could also be differentiated by examining their genitalia. For instance, the accessory sclerite in male *C. ferrugineus* is intricately connected to the two robustly sclerotized lobes located at the posterior end of the aedeagus, whereas other species like *C. capensis* exhibit relatively weaker sclerotization of these lobes. The sclerotization of the posterior lobes of the aedeagus in *C. ugandae*, while not as pronounced as in *C. ferrugineus,* is still visible [106]. Moreover, several researchers [107,108,109] proposed the identification of different species of *Cryptolestes* (*C. ferrugineus*, *C. pusillus, C. turcicus, C. pusilloides,* and *C. capensis*) based on the mitochondrial cytochrome c oxidase subunit I (COI) barcode region.

## 4. Biology and Development

### 4.1. Life Stages

*Cryptolestes ferrugineus* is holometabolous, which implies they undergo complete metamorphosis and consist of four life stages, namely egg, larva, pupa, and adult.

- Egg

The female *Cryptolestes ferrugineus* deposits eggs in small gaps in the grain kernels (under the outer layer of the seed coat), between the grain kernels, in small crevices or fractures in any structures, or in debris with the help of their substitutional ovipositor. Those caudal segments are generally retracted in the abdomen. During oviposition, those segments protrude out to facilitate the placement of the egg at a suitable location. The styli aid in the suitable orientation of the egg. Each female could lay about 200 to 500 eggs [111]. The eggs appear to be white and moderately translucent, with length and width in the range of 0.68 to 0.81 mm and 0.20 to 0.30 mm, respectively. The eggshell, after hatching, has a distinct iridescence [1].

b. Larva

Once the egg is ready to hatch, the larva breaks the eggshell (termed ‘chorion’) through a series of movements. The larva continuously produces those movements until its head emerges from the egg. Then, the larva crawls out of the eggshell with the help of its legs and a series of to-and-fro movements. Then, the larva starts its exploration of food. The larva mainly feeds on the germ portion of the wheat but also feeds on the endosperm during germ scarcity. The amount of food consumed depends on the environmental conditions. Under suitable conditions, the larva stays inside a kernel of wheat and forms a burrow through the consumption of wheat germ. It ejects the fecal material and molted exuviae through the opening created by the female adult during oviposition or by the larvae to enter the wheat germ [1]. The size of the larva ranges from 1 to 4 mm [111]. The average length of the larval stage varies under varying physical, environmental, and ecological stresses. For instance, the average length of *C. ferrugineus* larval stages was 56, 50, 36, and 21.8 days (d) in white flour, bran, wheat without germ, and wheat with germ, respectively [1].

There are four instars for *C. ferrugineus* larvae, which implies that they molt four times and become pupae after the fourth molting. The first, second, third, and fourth instar larval stages last about three to four, two to five, two to five, and five to eight days, respectively, at suitable conditions. The first instar larva is white in color, whereas the fourth instar larva becomes light tan in color. At the end of the abdomen of the fourth-instar larvae, caudal hooks are present, which aid in the backward movement of the larvae. The mouth parts of larvae and adults are similar. On evaluating the bioenergetics of *C. ferrugineus*, Campbell and Sinha [112] reported that the immature stages assimilated about 66% to 79% of the food consumed. They also reported that during development, the proportion of assimilated energy converted into tissue growth/biomass ranged from 3% (early first-instar larva) to 23% (older larva).

Before entering the next stage of development, the fourth-instar larva enters the burrow of the wheat and seals the burrow using debris and excrement through silken threads. Sometimes, they also pupate in other locations, such as crevices or the space between grain kernels. Two papillae, which are slightly and distinctly noticeable in the third and fourth instars, respectively, were reported to be responsible for the silk thread formation [1]. Compared with other *Cryptolestes* species such as *C. turcicus*, which can produce tough silk strong enough to produce a cocoon, *C. ferrugineus* forms fragile silk, which can only hold debris, bran, and excrement in place [113].

c. Pupa

Initially, the pupa is white, and over time, it turns into a light tan color with a triangular shape to some extent. The eyes of the pupae are dark brown in color [1]. The pupal stage lasts about three to six days at 32 °C and 75% relative humidity (RH).

d. Adult

**Figure 3:**
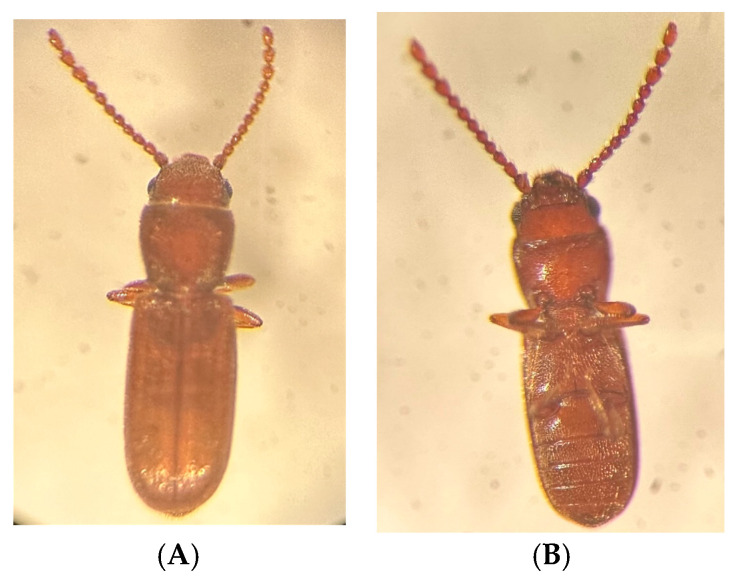
Dorsal view (**A**) and Ventral view (**B**) of *Cryptolestes ferrugineus* adult.

The adult that emerged from the pupa is light tan in color, which turns into a rusty brown color in one or two days (Figure 3). Immediately after emergence, the membranous pair of wings are stretched for a short duration, after which they fold beneath the elytra. The length of the adult is in the range of 1.70 to 2.34 mm, and the antennal length ranges from 0.70 to 1.14 mm [114]. A day or two after emergence, the adults start mating, the oviposition begins, and the cycle continues. The mean life span of adults ranges from 12 to 32 weeks (wk) depending on the density, feed, and sex ratio [115]. White and Bell [115] reported that the isolated virgin adults have a greater life span than the mated adults. The female:male sex ratio of *C. ferrugineus* adults was reported to be 1:0.64 [1] and 1:0.69 [116] on wheat and 1.1:0.8 on dates [59]. The longevity of female *C. ferrugineus* is longer than that of males [117]. Vendl et al. [118] studied the tarsal and inter-claw adhesive structures of *C. ferrugineus* using a scanning electron microscope and reported the following observations: (1) the length-to-width ratio of tarsi is about 9.5; (2) the first tarsomere is short and small (almost the same shape as the next tarsomere); (3) the last tarsomere is the longest among other tarsomeres; (4) the ventral side of the tarsomeres and the pre-tarsi do not have any adhesive structures; (5) a pair of apical setae on the unguitractor is present; (6) the lateral margin of the terminal tarsomere contains two pairs of setae, whereas the medial part of the ventral side of the margin is trapezoidal. The absence of adhesive structures in the tarsomeres is responsible for this species’ inability to climb inclined and smooth surfaces. The researchers compared the claw shapes of *C. ferrugineus* and *Oryzaephilus surinamensis* (L.) and reported that both species had similar claw shapes; however, the claws of *C. ferrugineus* were comparatively sharper and shorter (with a radius of 1.17 µm) than those of *O. surinamensis* (with a radius of 1.63 µm). This implies that *C. ferrugineus* has adapted its morphology to move over rough surfaces with smaller irregularities.

### 4.2. Sexual Dimorphism

**Figure 4:**
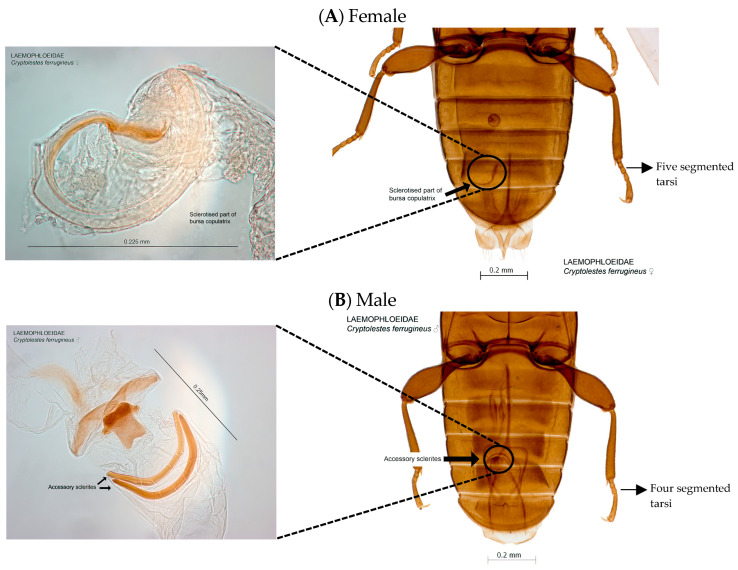
Female (**A**) and male (**B**) abdomens and genitalia of *Cryptolestes ferrugineus*. Image source: Pia Scanlon, adapted from Padil.gov.au under Creative Commons Attribution-Noncommercial 4.0 International license [121].

Male and female *C. ferrugineus* can be differentiated by observing their genitalia (Figure 4). The tarsi of female *C. ferrugineus* are all five-segmented (with tarsal formula 5-5-5), whereas those of males are four- and five-segmented (with tarsal formula 5-5-4). On the other hand, in females, the styli are present on the ninth abdominal segment, whereas they are absent in males. The male *C. ferrugineus* has a larger head and a wider thorax than the female [1]. A sex difference is observed in the mandibles. Precisely, the male mandible has a toothlike projection on the lateral ventral side near the base, while the female does not have the projection [1,119]. Chambers et al. [120] reported that the sexual differences of *C. ferrugineus* could also be identified based on the electroantennogram responses of the adults and showed greater electroantennogram amplitude in females than males towards the synthetic samples of the macrocyclic lactones containing aggregation pheromones.

### 4.3. Effects of Various Environmental Parameters on the Biology of Cryptolestes ferrugineus

The development time, oviposition rate, and life span of the adults depend on various environmental, physical, and ecological factors such as temperature, RH, availability of food, type of food, pesticide exposure, presence of predators or parasitoids, and genetics. For instance, during the first 30 d of adult life at 30 °C and 70% RH, the average oviposition rate of *C. ferrugineus* females is 7.5 and 5.6 eggs/d in flour and wheat kernels (moisture content 16 to 18%) consisting of 3% (weight basis) flour, respectively [122]. At most temperatures, temperature has the highest relative influence on insect development, followed by moisture and diet; near the optimal temperature, moisture and diet have a stronger effect on larval development than temperature [123]. Table 3 lists the development period of *C. ferrugineus* at various temperatures, RH, and food sources.

#### 4.3.1. Temperature

Al-Salihi and Al-Azawi [59] reported that the duration is 3.2 d for eggs with a hatching rate of 96.8%, 70.3 d for larvae, 3.6 d for the pre-pupal stage, 6.1 d for the pupal stage, and 186 d for adults at 30 °C and 70% RH. One female can lay 558 eggs. The developmental temperature and RH range from 20 to 40 °C and 40 to 95%, respectively [3]. The optimal temperature and RH are 33 °C and 70 to 80%, respectively [3], whereas the preferred temperature (the temperature towards which the insects move) is 30 to 36.5 °C [126]. *Cryptolestes ferrugineus* can also develop at temperatures ranging from 20 to 42.5 °C [127]. The intrinsic rates of natural increase of *C. ferrugineus* were the highest at 35 °C and 90% RH and the lowest at 20 °C and 70% RH [128]. At 42.5 °C, larval and pupal mortality were 98% [128]. *Cryptolestes ferrugineus* is one of the most cold-tolerant species, with the adult being the most cold-hardy stage [129]. *Cryptolestes ferrugineus* adults, after being acclimated to temperatures of 18, 10, and 5 °C for one week at each temperature, took about 58 d at −10 °C to reach 95% mortality, whereas *C. turcicus* and *C. pusillus* reached 95% mortality at 39 and 11 d, respectively [130]. At temperatures below 23 °C, the rate of reproduction decreases; at temperatures below 21 °C, the insects cannot fly [109]. On the other hand, Cox and Dolder [131] reported that the minimum temperature for *C. ferrugineus* flight was 20 °C, whereas in laboratory-cultured strains for a period of over 20 years, one insect was reported to fly at 17.5 °C. *Cryptolestes ferrugineus* does not lay eggs below 17.5 °C [128]. Under suitable environmental conditions (at 32 °C and 75% RH), the eggs hatch in three to four days [1]. Eggs do not hatch below 15 °C [132]. Ashby [133] reported that the rate of respiration and development of *C. ferrugineus* proportionally increased with the rise in temperature, in the range of 21 to 33 °C. The development rate of *C. ferrugineus* eggs is linearly related to temperature (T) (Egg development rate, D = 0.0169–0.258 T) [132].

Temperature is one of the main factors that influences the population dynamics of *C. ferrugineus* [134]. An extensive review of the application of temperature to control stored product insects is available [135]. At −10 °C, the LT_50_ (lethal time for 50% of a population) values for egg, young larva, old larva, pupa, and adult were reported to be 8, 4, 16, 11, and 91 h, respectively [130].

##### Acclimation

Acclimation is one of the important parameters that determines the cold tolerance levels of insects, in addition to influencing their behavior, survival, growth, and multiplication. In cold-acclimated *C. ferrugineus*, trehalose and amino acids including proline, asparagine, valine, lysine, leucine, isoleucine, alanine, phenyl alanine, glutamic acid, and aspartic acid, as well as phosphoethanolamine (a phospholipid precursor), were higher than in unacclimated *C. ferrugineus* [136]. Furthermore, the acclimation increased the mean fresh weights of *C. ferrugineus* [137]. The acclimation temperature was found to affect the behavior of *C. ferrugineus* more than the exposure time [138]. When acclimated to low temperatures (15 to 5 °C) for some time, most stored-product insects were found to increase their cold tolerance by 2 to 10 times [135]. The acclimated *C. ferrugineus* was reported to be more cold-hardy than the non-acclimated ones [139]. Precisely, *C. ferrugineus* acclimated at 15, 10, and 5 °C consecutively for two weeks at each temperature had LT_50_ and LT_90_ (lethal time for 90% tested individuals) of 24 and 42 d, respectively, at −10 °C, whereas unacclimated *C. ferrugineus* had LT_50_ and LT_90_ of 1.4 and 2.7 d, respectively, at the same temperature [136]. Smith [129] reported that the LT_50_ values of acclimated adults increased by 9 and 56 times, respectively, at −6 and −12 °C compared with the unacclimated adults. In addition, the supercooling points of *C. ferrugineus* adults were −16.5, −20, and −21 °C for unacclimated, acclimated at 15 °C, and acclimated at 15 °C followed by acclimation at 4 °C, respectively. The mean survival times of *C. ferrugineus* directly transferred to 9 °C from warmer temperatures (30 or 32 °C) were 4.3 weeks, whereas the mean survival times of those acclimated (about 4.5 °C/week) to 9 °C were 7.6 weeks [140]. Burks and Hagstrum [141] examined the rapid cold hardening ability of five different species (*C. ferrugineus*, *O. surinamensis*, *Rhyzopertha dominica* (Fabricius), *Sitophilus oryzae* (L.), and *Tribolium castaneum* (Herbst)) and reported that *C. ferrugineus* is more capable of rapid cold hardening than other tested species.

#### 4.3.2. Moisture Content

Damper grains facilitate more convenient feeding than dry grains for *C. ferrugineus*. The development of *C. ferrugineus* is limited when the moisture content of the grain or RH is below 12% or 40%, respectively [111]. The intrinsic rates of natural increase of *C. ferrugineus* were almost the same at 70 and 90% RH, whereas they were the lowest at 40% RH [128]. Similarly, Evans [140] reported shorter insect survival at 45% RH than at 70% RH. Throne [142] studied the progeny of *C. ferrugineus* at different moisture contents (11.3, 12.4, and 14.8% at 43, 56, and 75% RH, respectively) and reported that the number of offspring produced on damaged grain increased linearly with moisture content. Similarly, Throne and Culik [143] reported that the corn maintained at 75% RH showed higher progeny production and lower development time for *C. ferrugineus* when compared with those at 43% RH. Bishop [117] reported that the egg production and longevity of *C. ferrugineus* increased with an increase in RH. However, compared with *C. minutus* and *C. turcicus*, *C. ferrugineus* was less sensitive to 40% RH at 32.2 °C [117]. Kawamoto et al. [132] studied the mortality and development of *C. ferrugineus* eggs at different RH (50, 60, 70, 80, and 90%) and reported that RH does not affect the mortality and development of eggs. With an increase in temperature from 25 to 35 °C, the effect of RH on *C. ferrugineus* rate of oviposition was reported to be more pronounced [128]. *Cryptolestes ferrugineus* adults preferred to lay eggs in damper grain (18% moisture content) to drier grain (14% moisture content) [144]. They preferred the drier region (70% RH) to the moister region (85% RH) in the absence of grain, whereas within a grain bulk, adults accumulated in the pockets of damp grain [144].

#### 4.3.3. Diet

Although *C. ferrugineus* primarily feeds on germ and is considered a secondary pest, it is capable of infesting grain kernels with broken seed coats that are present in a sound grain mass [3]. The type and quality of food greatly influence the survival, growth, and multiplication of *C. ferrugineus.* However, certain studies found contradictory results regarding the suitability of diets for *C. ferrugineus*. Larvae of *C. ferrugineus* have better survival and faster development in a wheat kernel with a germ than those without a germ or on bran or white flour [1]. Moreover, the oviposition rate on whole-wheat flour was greater than wheat kernel at all tested densities (4, 16, and 64 pairs per vial), except for one pair per vial [145]. Tuff and Telford [146] reported that *C. ferrugineus* was not able to invade sound kernels, whereas it could infest seeds with damaged grain coats. Similarly, Throne and Culik [143] reported higher progeny production and decreased development duration on cracked corn compared with undamaged kernels. However, the level of cracking on the corn did not significantly affect the survival of the immature stages of *C. ferrugineus* [147]. Shufran et al. [148] performed a laboratory experiment on the host suitability of pecan and wheat for various stored-product insects and reported that *C. ferrugineus* were observed to produce more immatures on unsorted pecan, cracked pecan, and nutmeats than on in-shell pecan; however, only fewer adults were observed on different types of pecans than wheat. This implies that pecans lack certain dietary requirements for *C. ferrugineus*. White and Loschiavo [149] reported that the slower developmental time and higher larval mortality of *C. ferrugineus* on oats compared with wheat were due to the nutritional insufficiency and unpalatability of oats. Even though *C. ferrugineus* can survive on hemp seed and its dockage, it does not flourish [150]. Also, *C. ferrugineus* prefers wheat kernels as compared with canola and rapeseed [151]. Durum Kyle, Coulter, and Medora are suitable wheat varieties for the oviposition and development of *C. ferrugineus* [149]. Jagadeesan et al. [152] evaluated the suitability of nineteen grain-based diets on the number of live adult progeny developed and concluded that diets containing (a) barley flour, (b) rolled oats and cracked sorghum, (c) wheat flour and barley flour, and (d) cracked sorghum alone resulted in higher progeny production of laboratory strains, whereas diets containing (a) rolled oats and cracked sorghum, (b) wheat flour and barley flour, and (c) barley flour alone were suitable for field-collected strains. They also reported that diets containing cracked sorghum were better than those containing cracked maize or wheat. The reason might be that the laboratory strain used was cultured in a diet containing rolled oats, cracked sorghum, and yeast for five generations prior to the experiment; furthermore, the insects were collected from stored sorghum. They hypothesized that the literature published on the successful culturing of *C. ferrugineus* on corn [143,147] could have been collected from stored maize. The diet also influences the cold tolerance of *C. ferrugineus.* For instance, the LT_50_ of *C. ferrugineus* adults at −10 °C in grain, flour, and Brewer’s yeast and flour alone were 104, 79, and 42 h, respectively, and the supercooling points were −20.6, −22.9, and −19.4 °C, respectively [130].

*Cryptolestes ferrugineus* feeds on certain fungal species as supplementary or alternative food sources. Nevertheless, in the absence of grain, *C. ferrugineus* feeds mainly on fungi. Sinha [153] reported that *C. ferrugineus* completed its development on 10 species of fungi (*Absidia orchidis* (Vuill.) Hagem, *Alternaria tenuis* sensu Wiltshire, *Curvularia tetramera* (McKinney) Boedijn, *Fusarium moniliforme* Sheld, *Helminthosporium sativum* P., K., and B., *Mucor sphaerosporus* Hagem, *Nigrospora sphaerica* (Sacc.) Mason, *Penicillium cyclopium* Westl., *Stemphylium botryosum* Wallr, and *Trichothecium roseum* Lk., among 23 species tested. The shortest and longest developmental periods were about 22 and 34 d, respectively, on *T. roseum* and *F. moniliforme*. Similarly, Loschiavo and Sinha [154] studied the oviposition, feeding, and aggregation of *C. ferrugineus* in the presence of different species of seed-borne fungi and revealed that *N. sphaerica*, *M. sphaerosporus*, *Hormodendrum cladosporiodes* (Fres.) Sacc., and *C. tetramera* were the most suitable fungi for oviposition and feeding. The differences in responses of *C. ferrugineus* were observed for different species from the same genus. For instance, *P. terrestre* was not suitable for oviposition and feeding, whereas *P. cyclopium* and *P. funiculosum* were moderately suitable. On the other hand, *C. ferrugineus* was observed to feed moderately on *Aspergillus flavus* and did not lay eggs; however, they were observed to lay a few eggs and feed slightly on *A. fumigatus*. Aggregation of *C. ferrugineus* was observed on grain kernels containing mycelia and spores of *N. sphaerica* [154].

Overall, *C. ferrugineus* can feed on more than 65 commodities, including but not limited to wheat, paddy, sorghum, barley, flax, black pepper, cocoa bean, coffee bean, cassava root, palm kernel, peanut, chili, hemp, sunflower seed, oat, bamboo leaf (dried), bark, animal feed, beam cake, wheat flour, wheat product, barley (pearl), yam, rice, cashew, raisin, date, fennel seed, fig, broad bran, cassava root flour, chili pod (dried), peanut product, soybean paste, and vegetable (preserved) [155], but do not actively multiply on products such as wood, fiber, and textile.

#### 4.3.4. Insect Density

Crowding plays a significant role in the population dynamics of *C. ferrugineus* since crowding can encourage fighting and cannibalism, which results in high egg damage and high mortality [134]. At 30 °C and 70% RH, the number of eggs produced per female per day was 6.4 and 1.5 when 1 and 64 pairs of adults, respectively, were present in a vial containing 0.5 g flour, whereas in a 1 g wheat kernel, the number of eggs produced per female per day was 5.6 and 0.75 in the presence of 1 and 64 pairs of adults, respectively [145]. Development times (egg to adult) on 0.5 g flour were 24 and 87.1 d, with an initial larval count of 1 and 32, respectively, per vial. Smith [145] also found that the mortality of the insects increased with density. White and Bell [115] reported that the amount of energy outflow and the physical injury during copulation affect the survival of insects at different densities and at different sex ratios. Studies on the population dynamics of *C. ferrugineus* revealed that the population dynamics of the species are influenced by patch size and temperature [134,156]. Moreover, they also reported that the total insect number and kernel infestation percentage were positively correlated. All these studies concluded that density affects the oviposition, development, and mortality of *C. ferrugineus.*

## 5. Ecology and Behavior

### 5.1. Refuge-Seeking Behavior

Some of the review articles covered the refuge-seeking behavior of stored grain insects [157,158]. Refuge-seeking behavior is the ability of the stored grain pests to hide in the structural cracks and crevices of the storage structure, which contain grain residues. The refuge provides food and shelter to the insects, in addition to protecting them from insecticide treatments. The hidden insects emerge and reinfest nearby grain when the conditions are favorable. Even in the absence of food, *C. ferrugineus* was reported to be attracted to the refuge, possibly for the physical contact around their bodies. This could also be the reason for their occurrence near the container boundary during laboratory experiments [159].

On analyzing the samples obtained from structural cracks and surfaces from 34 empty storage structures in the Prairie provinces of Canada (Manitoba, Saskatchewan, and Alberta), *C. ferrugineus* was identified in 36% of the sampled structures [29]. The effects of different temperatures, refuge contents, food availability, and different strains on the refuge-seeking behavior of *C. ferrugineus* have been evaluated by Cox et al. [160] and Cox and Parish [159]. Cox et al. [160] observed the refuge-seeking behavior of different strains of *C. ferrugineus* at different temperatures (15, 20, 25, and 30 °C) and reported that about 45% and 20–30% of the insects were found to remain inside the refuge at the end of 2 wk for *C. ferrugineus* strains that were reared in the laboratory for over 25 years (yr) and those obtained from grain stores and mills in the UK, respectively. Moreover, they also observed that the refuge-seeking behavior of different strains of *C. ferrugineus* varied with varying temperatures. The refuge-seeking behavior of *C. ferrugineus* females was greater than that of males, and that of adults 0–3 wk old was greater than that of 10–12 wk and 16–18 wk old adults [161]. This was because the refuge would have attracted females for oviposition and younger adults since oviposition is greater in younger adults than older ones [128].

### 5.2. Flight Activity

The flight activity of insects determines their ability to infest the stored grains in different bins. The level of infestation inside a grain bin varies with the number of insects immigrating into the bin. The flight activity of *C. ferrugineus* depends on external factors such as air temperature, wind direction, wind speed, and day length [162,163]. During a flight activity study of *C. ferrugineus* in southern New South Wales, Australia, Holloway et al. [163] observed no flight activity during the winter months (June, July, and August). *Cryptolestes ferrugineus,* captured on glue boards installed in and around the warehouses of Kansas and Nebraska, U.S., reached a peak in early September and declined through early November [164]. Hagstrum [165] studied the immigration of insects in 34 bins with varying capacities (36 to 238 t) containing hard red winter wheat on 12 farms from July to December 1998 in Kansas, U.S., and found the immigration of *C. ferrugineus* in all the 34 bins. The drop in immigrated insect count was reported when the ambient temperature dropped below 20 °C. Thus, *C. ferrugineus* shows seasonal variation in flight activity and immigration. This is because the minimum temperature for their flight initiation is 20 °C [131]. Hagstrum [99] observed the distribution of *C. ferrugineus* on three farms in Kansas, U.S., and reported that most of the *C. ferrugineus* infestation occurred after the grain was loaded into the bin. In addition, the number of insect counts decreased in the top layers. Hagstrum [99] concluded that *C. ferrugineus* adults fly to the top of the bin and then distribute it to other parts of the grain inside the bin.

### 5.3. Mating Behavior

Male and female adults of *C. ferrugineus* start mating within one or two days after they emerge. When a male identifies a potential female, the male adult turns and follows the female. Boukouvala et al. [119] performed an experiment to evaluate the lateralization of males during courtship and mating and reported that most (41%) *C. ferrugineus* males showed a left-biased approach (turning 180° to their left) towards females, whereas 34%, 14%, and 11% approached females from the right side, back side, and front side, respectively. Moreover, they also revealed that the left-biased males showed shorter durations of mate recognition and chasing as well as lower copulation attempt durations, with higher successful mating attempts compared with the right-biased males. The male follows the female by nudging the tip of the female’s abdomen with the male’s head. Once the female stops, the male strokes the female elytra with its antenna. The male continues its efforts to succeed by crawling on the back of the female and turning. Only the flickering of the female’s antenna was reported during the process. Once the male and female are coupled, the first copulation was observed to last for 105 min, followed by separation for 20 min. Then, the second and third copulations were observed for 35 and 95 min, respectively. During coition, the male and female are firmly attached since the aedeagus is inserted deeply into the female’s genital tract [1].

### 5.4. Chemical Ecology

#### Pheromones

Pheromones are chemical substances produced by insects that affect the behavior of other individuals of the same or other species. *Cryptolestes ferrugineus* males produce pheromones, namely (E, E)-4,8-dimethyl-4,8-decadien-10-olide (ferrulactone I) and (3Z,11S)-3-dodecen-11-olide (ferrulactone II) [166]. The pheromones are produced in the alimentary canal and/or the Malpighian tubules. Researchers have shown the possibility of isolating the aggregation pheromones (ferrulactone I and II) from *C. ferrugineus* [167]. The naturally produced ratio of ferrulactone I to ferrulactone II by *C. ferrugineus* was 1.6:1.0 [168]. Adults of mixed sex and age responded to the odor of mixed-sex adults, frass, pentane extracts of frass, and Porapak Q-captured volatiles from adults or frass [166]. Moreover, they also observed responses from both sexes to the volatiles in males. Similarly, Currie et al. [169] reported that in the absence of air currents and food for feeding, male and female *C. ferrugineus* were attracted to a single male in an apparatus of 10 cm length; when grain was present, a single male was not enough to attract *C. ferrugineus*. However, a significant number of females were attracted to 50 males.

According to Oehlschlager et al. [170], the aggregation pheromones produced by *C. ferrugineus* can act alone as well as synergistically. Moreover, the *C. ferrugineus* species is not cross-attracted to the pheromones produced by other species such as *Oryzaephilus mercator*, *O. surinamensis*, *C. turcicus*, and *C. pusillus* (Schonherr) [170]. Chambers et al. [120] analyzed the electroantennogram (EAG) responses of the males and females of *C. ferrugineus* and reported that females produced EAG with higher amplitude. Thus, the greater response of females to the pheromones produced by males implies the importance of pheromones in mate identification and courtship. While determining the flight activity of *C. ferrugineus* in farms in south-eastern Australia, Holloway et al. [163] reported that more females were trapped in traps with pheromone (female:male ratio of 3:1), whereas in passive trapping, the female:male ratio caught was 1:1. Similarly, during a seasonal flight activity study at grain storage sites in South Carolina, U.S., Throne and Cline [100] observed more *C. ferrugineus* females at all the tested sites. These results further confirm the higher attraction of females towards the pheromone.

### 5.5. Heat Production

Heat production of 4 wk old adults and second, third, and fourth instar larvae was in the range of 0.72 to 21.47 µW/insect and 0.37 to 17.53 µW/larvae, respectively, at the tested temperatures (15, 20, 25, 30, and 35 °C) and moisture contents (12, 15, and 18% wet basis) [171,172]. The maximum rate of heat production was observed in adults over the age of 4 wk. Cofie-Agblor et al. [171,172] also found heat production of adults: (1) varied with insect density; (2) exponentially increased with increase in temperature from 15 to 35 °C; (3) increased with increase in moisture constant; however, the rate of increase from 15 to 18% was lower than that from 12 to 15%; and (4) increased with increase in level of wheat breakage; however, the rate of increase from 10 to 20% breakage was lower than that from 0 to 10%.

*Cryptolestes ferrugineus* multiplication is associated with grain heating; however, at low density (less than five adults/kg), they cannot initiate heating [173]. Smith [174] added water to increase the moisture content of wheat stored in a metal granary and reported that the increase in moisture content led to the heating of the grain as well as a rapid increase in the *C. ferrugineus* population. Thus, the high multiplication of *C. ferrugineus* is a consequence of grain heating and not the cause.

### 5.6. Movement and Distribution Inside Grain

The movement of insects inside grain can be random (non-directional) or biased (non-random or directional). Under uniform environmental conditions, insects tend to wander inside the grain randomly and reach biologically suitable locations. On the other hand, individual insects move in a non-random direction in search of food, refuge, a mating partner, to escape predators, or due to non-uniform environmental conditions in stored grain structures [158]. The biased movement is also influenced by pheromones, host stimuli, the presence of other organisms such as parasitoids and predators, and other physical stimuli such as temperature, moisture content, gas concentration, dockage, foreign materials, light, and radiation [175]. The tendency of the insects to move towards a particular environment could be due to (a) behavioral response to physical stimuli, (b) physical response, wherein the rate of their metabolic activities changes at different environments, and (c) survival response, wherein the insects avoid extreme temperatures unsuitable for their survival, growth, and multiplication [175]. More detailed information on the factors influencing the movement and distribution of *C. ferrugineus* [2] and other stored-product insects [176] is available elsewhere. From those review articles, it can be noted that *C. ferrugineus* detects cues and resources and tends to move towards an environment for their growth and multiplication. For instance, *Cryptolestes ferrugineus* adults prefer warmer grain to their surrounding cooler grain in the absence of other factors, and the adults could detect the temperature gradient in less than 1 h [177,178]. They detected a temperature difference of 1 °C in 24 h in a tested cylinder of diameter 56 cm and height 9 cm [177]. *Cryptolestes ferrugineus* prefer damp and mold-infected grain rather than dry grain since damp grain is soft and easy to oviposit into, and they could feed on the mold itself [144]. While determining the spatial and temporal distributions of *C. ferrugineus* adults inside a grain bin containing 1.5 t of wheat, Jian et al. [179] reported that the level of aggregation decreased with an increase in insect density. This is because at lower density, aggregation would increase their possibility of meeting mating partners. White et al. [180] reported that *C. ferrugineus* adults prefer to move towards elevated carbon dioxide levels, whereas prolonged exposure to higher levels of carbon dioxide is lethal to the insects.

The one- and two-dimensional (D) movements of *C. ferrugineus* have been extensively studied at various environmental conditions, such as temperature, moisture contents, and their gradients, in the laboratory [126,178,181,182,183]. Recently, Bharathi et al. [184] developed an experimental setup consisting of 343 metal-based mesh cubes arranged inside a wooden box to study the movement of insects in three dimensions. The researchers concluded that the 3D movement and distribution patterns were similar to those in 1D and 2D [184,185]. However, the 3D movement of *C. ferrugineus* was observed only in uniform environmental conditions. Bharathi et al. [186] observed the movement and distribution of *C. ferrugineus* inside a grain bin filled with 300 t of wheat for 26 months in Winnipeg, Canada. They reported that *C. ferrugineus* inside the grain bin followed a movement and distribution pattern similar to those reported in the laboratory experiments under similar environmental conditions. The activity of *C. ferrugineus* inside a 300 t wheat grain bin was reported to reduce near the boundary when the temperature dropped during the winter (especially when the temperature dropped below 2.5 °C) and resume when the temperature increased above 4.5 °C [186,187].

Briefly, temperature gradients and moisture differences are the predominant factors that influence the movement and distribution of *C. ferrugineus*, whereas the presence of mold, type of food, dockage, intergranular grain spaces, and ventilation are trivial factors that influence the movement and distribution of *C. ferrugineus* adults [2]. Based on limited research, the carbon dioxide gradient also seems to be a significant factor [180], but more research is required on the movement of insects under carbon dioxide gradients. Thus, the behavior of *C. ferrugineus* is the result of exploration for a location that is biologically suitable and physically comfortable for their survival, growth, and multiplication.

## 6. Interaction with Other Organisms

### 6.1. Interspecific Interaction

The interaction of *C. ferrugineus* with the following stored grain insects in the laboratory has been studied: *C. turcicus*, *C. pusillus*, *T. castaneum*, *Lasioderma serriocorne*, and *S. oryzae* (Table 4). Few studies explored the interspecific interaction of different stored-product insect species in field conditions. For instance, Nansen et al. [188] sampled wheat from 129 grain silos in Kansas in 1999–2001, analyzed the densities of *R. dominica*, *C. ferrugineus,* and *T. castaneum,* and reported the intra- and inter-specific interactions of the insects. The researchers observed that the presence of *C. ferrugineus* reduced the density of both *R. dominica* and *T. castaneum*. However, the populations of *T. castaenum* and *R. dominica* did not influence the insect count of *C. ferrugineus*. These studies imply that the interspecific interaction of species depends on various factors such as the availability of food, cannibalistic behavior, environmental conditions, whether the insect is a primary or secondary feeder, and its predative nature.

### 6.2. Nature Enemies

Wasps such as *Habrobracon hebetor* (Say) (Hymenoptera: Braconidae)*, Cephalonomia waterstoni* (Gahan) (Hymenoptera: Bethylidae)*, Brachymeria* sp., *Anisopteromalus calandrae* (Howard) (Hymenoptera: Pteromalidae), *Lariophagus distinguendus* (Förster) (Hymenoptera: Pteromalidae), *Holepyris sylvanidis* (Brèthes) (Hymenoptera: Bethylidae), and *Theocolax elegans* (Westwood) (Hymenoptera: Pteromalidae), have been identified as parasitoids on stored grain pests and are used as biological control agents [73,155,192,193]. Among these, the most common parasitoid of *C. ferrugineus* is *C. waterstoni* [155]. Adult females of *C. waterstoni* paralyze, feed, and oviposit on their hosts. *Cryptolestes ferrugineus* larvae paralyzed by *C. waterstoni* cannot advance into the next developmental stage, and as a result, those larvae are available as oviposition sites for a minimum of 2 wk [194]. *Cephalonomia waterstoni* has been identified as one of the best biological control agents because it effectively follows the kairomonal trail inside the grain [195], has the ability to feed on all larval instars, has a generation time half that of the host, and is extremely host-specific [194]. Flinn and Hagstrum [196] developed a model to predict the phenology of *C. waterstoni* and *C. ferrugineus* in relation to grain temperature and reported that the effect of the parasitoid on the host is the highest when released during the first production of fourth-instar larvae. The combined application of an insecticide and a parasitic wasp could result in effective control of *C. ferrugineus*. Flinn et al. [197] reported that the combined application of transgenic avidin maize powder and the parasitoid wasp *T. elegans* drastically reduced the population of *C. ferrugineus* in maize compared with *T. elegans* alone, even though *C. ferrugineus* did not grow well in maize samples as compared with other insect species considered in this study such as *S. zeamais* and *T. castaneum*.

Mites such as *Acarophenax lacunatus* (Cross and Krantz) [198] and *Cheyletus eruditus* (Schrank) [199] have been identified as biological control agents of *C. ferrugineus*. *Acarophenax lacunatus* was observed to prey on the eggs and reduce the larval population of *C. ferrugineus*. Thus, these parasitic mites were able to successfully reduce the instantaneous rate of *C. ferrugineus* increase [200].

Entomopathogenic fungi such as *Beauveria varroae*, *B. bassiana,* and *Purpureocillium lilacinum* were found to be associated with *C. ferrugineus* in wheat and maize samples in Central and South Anatolia in Turkey [92]. Similarly, Wakil et al. [80] explored the naturally occurring entomopathogenic fungi infecting stored grain insects in Punjab, Pakistan, and reported fungal species such as *Alternaria alternata*, *A. solani*, *Aspergillus flavus*, *A. fumigatus*, *A. parasiticus*, *A. niger*, *Bipolaris oryzae*, *C. lunata*, *Fusarium oxysporium*, *Helminthosporium oryzae*, *P. capsulatum*, *P. chrysogenum*, *Phomopsis* sp., and *Rhizopus stolonifers.* Among the insect species tested, *C. ferrugineus* (0.1% occurrence) was less affected by entomopathogenic fungi than *Tribolium castaneum* (Herbst) (0.3% occurrence) and *Sitophilus oryzae* (L.) (0.2% occurrence). However, *C. ferrugineus* eggs are resistant to *B. bassiana* infection [201]. In 1988, Isabelle I. Tavares identified an undescribed fungal parasite species in the genus *Dimeromyces* (Ascomycetes: Laboulbeniales) on the last visible abdominal segment near the ovipositor base of *C. ferrugineus* [202]. Lord et al. [203] reported that *Nosema oryzaephili* microsporidia at 10^6^ spores/g of diet resulted in about 99% infection of *C. ferrugineus* larvae after three weeks of exposure.

Ünal and Koçak [204] reported the association of endosymbionts such as *Wolbachia, Rickettsia,* and *Spiroplasma* with *C. ferrugineus*. *Mattesia oryzaephili* and *M. dispora* were reported to be two of the pathogens that infect *C. ferrugineus*. Lord [205] reported that at a dose rate of 10^5^ oocysts/g of diet, the mortality and infection rates of the fourth instar of *C. ferrugineus* were higher with *M. oryzaepili* than with *M. dispora*. Thus, the presence of these two *Mattesia* species could lead to a decline in *C. ferrugineus* populations.

## 7. Mathematical Models Developed

Several mathematical models, focusing primarily on the population dynamics and movement behavior of *C. ferrugineus*, have been developed. Some of the models are listed in Table 5. These models highlighted the importance of factors such as temperature, moisture content, and the distribution pattern of *C. ferrugineus* within the storage facilities. The models also considered the effects of various environmental conditions on the survival, development, and multiplication of *C. ferrugineus*, providing insight for pest management strategies. To enhance the accuracy of predictions, a few models emphasized the incorporation of feedback mechanisms, heat production, and insect movement. Thus, these models help us understand the complex interactions between *C. ferrugineus* and its environment, facilitating the development of effective pest management strategies.

## 8. Directions for Future Research

Despite the significant progress made in understanding the biology, ecology, and behavior of *C. ferrugineus*, complete knowledge on several aspects of this species is still lacking. The following studies could be considered avenues for future research:

- Studies on the genetic basis of traits related to *C. ferrugineus* ecology and behavior, such as resistance to insecticides and reproductive behavior.
- Understanding the specific mechanism responsible for the adaptability of *C. ferrugineus* to various environmental conditions.
- Interspecific interaction of *C. ferrugineus* with other insects and organisms in storage facilities and in the wider landscape.
- Identification and development of effective and sustainable management strategies to control the spread and multiplication of *C. ferrugineus.*
- Investigation of the role of microbes in the ecology, behavior, and control of *C. ferrugineus.*
- Application of molecular markers and population genetic approaches to understand the phylogeography and evolutionary history of the insect.
- Development of integrated management strategies under climate change conditions involves the integration of knowledge from various aspects such as ecology, behavior, biology, and economics.
- Development and validation of mathematical models that consider time-dependent spatial distributions of temperature, moisture, CO_2_, and biological agents such as insects and molds on insect numbers throughout the grain mass.

## 9. Remarks

A large number of studies performed by researchers around the world on the ecology and behavior of *C. ferrugineus* demonstrate the global importance of this species. Its ability to thrive in a wide range of environments and feed on a variety of food sources highlights its significance in the ecosystem. The complex behavior of *C. ferrugineus* adds to its uniqueness and highlights the importance of further research in the area. However, with the increasing impact of environmental factors such as climate change and resistance to insecticides, it is significant to explore the potential impact of *C. ferrugineus* populations and their role in the ecosystem and develop a sustainable pest management strategy to control the population. Thus, this study aids in understanding the biology, ecology, and behavior of *C. ferrugineus* and provides a foundation for the development of sustainable practices to ensure the preservation of biodiversity moving forward.

## Figures and Tables

**Table 2:** Identification of different species of *Cryptolestes* [104,110].

Part	*Cryptolestes ferrugineus*	*Cryptolestes pusillus*	*Cryptolestes turicicus*
Antennae	Subequal in male and female; half as long as the body	Longer in males than females; two-third length as their body	Longer in males than females; equal to or longer than their body
External mandibular tooth	Present in male	Absent in male	Absent in male
Head	Transverse ridge near dorsal posterior margin is absent	Transverse ridge near dorsal posterior margin is present	Transverse ridge near dorsal posterior margin is present
Pronotum	Narrowed posteriorly, especially in males	Transverse, slightly narrowed posteriorly in males	Nearly quadrate
Number of rows of setae between first and second and between second and third elytral striae	Four	Four	Three

**Table 3:** Development period of *C. ferrugineus* at different temperatures, relative humidities, and food sources.

Food Source	Temperature (°C)	Relative Humidity (%)	Development Period (Days)	References
Whole maize grain	32 ± 1	75 ± 2	51.20	[124]
Broken maize			58.40	
Maize flour			56.10	
Ground wheat: Germ (4:1, *w*/*w*)	30 ± 1	75 ± 5	28.05	[125]
Wheat germ	21.1	75	64.2	[1]
	26.7		27.4	
	32.2		20.5	
	37.8		21.0	
	32.2	50	32.41	
		65	26.60	
		75	21.70	
		90	20.84	
		100	20.33	
Half-kernel of wheat split longitudinally, and a portion of germ remained on each piece	32.2	60	27.0	[117]
		70	27.5	
		80	24.0	
		90	23.0	

**Table 4:** Interspecific interaction of *C. ferrugineus* with other stored grain insects.

Insect	Grain	Experimental Conditions	Observation/Conclusion	Reason	References
*Tribolium castaneum*	Wheat and wheat feed	Temperature: 30 °C RH: 60%	*C. ferrugineus* restricted the survival of *T. castaneum.*	The immature stages of *C. ferrugineus* stay hidden under the seedcoat, making it difficult for *T. castaneum* to discover them and prey on them, whereas *C. ferrugineus* preys effectively on the exposed stages of *T. castaneum,* such as eggs, larvae, and pupae [189].	[190]
	Ground wheat	Temperature: 25 and 30 °C RH: 70%	Both species were cannibalistic in nature; however, *T. castaneum* adults were more effective cannibals than *C. ferrugineus.*	*T. castaneum* adults (weighing about 2 mg) were larger in size than *C. ferrugineus* (weighing about 0.2 mg).	[189]
	Wheat	Insect densities: 250, 500, and 1000 adults/kg Moisture contents: 12% Temperature: 30 °C	Significantly lower *C. ferrugineus* adult population when reared alone, as compared with that of the combination, at all the tested densities.	The smaller-sized first-instar larvae of *C. ferrugineus* could have had difficulty penetrating the wheat germ in dry grain, whereas, in combination, *T. castaneum* could have damaged the grain and made it easier for *C. ferrugineus* larvae to enter and feed.	[191]
		Insect densities: 250, 500, and 1000 adults/kg Moisture contents: 15% Temperature: 30 °C	*C. ferrugineus* reared alone had a higher adult population than those in combination.	In damp grain, the larvae would have penetrated easily, and the adults would have fed well.	
*Cryptolestes turcicus*	Wheat feed	Temperature: 27.5 °C RH: 90%	*C. turcicus* adult survival in the presence of *C. ferrugineus* depended on the initial number of *C. turcicus*, while the survival of *C. ferrugineus* depended majorly on the environment.	In the presence of *C. turcicus* larvae at higher density, *C. ferrugineus* show delayed induction of pupation and in some cases, the delay in pupation could lead to loss in its pupation ability. Moreover, the metamorphosing stage of *C. ferrugineus* is susceptible to cannibalism since their cocoon contains little silk and is fragile, whereas *C. turcicus* forms a tough silk cocoon and is protected inside it.	[113]
*Cryptolestes pusillus*	-		*C. pusillus* was attracted to the pheromones of *C. ferrugineus.*	-	[120]
	Cracked wheat or cracked maize	Temperature: 20, 25, 30, and 35 °C RH: 70% Insect density: 20 adults/100 g	*C. ferrugineus* multiplied better at warmer temperatures (30 and 35 °C on wheat and 35 °C on maize), whereas *C. pusillus* multiplied better at colder temperatures (20 °C).	Optimal developmental temperature range for *C. ferrugineus* is 20 to 42.5 °C, whereas for *C. pusillus*, it is 17.5 to 37.5 °C. At warmer temperature (35 °C), the egg production and development rate of *C. ferrugineus* are at their maximum. Moreover, the mortality of *C. pusillus* at warmer temperature (35 °C) was higher than that of *C. ferrugineus.*	[127]
*Sitophilus* *oryzae*	Wheat and wheat feed	Temperature: 30 °C RH: 60%	The presence of *C. ferrugineus* in wheat inhibited the growth of *S. oryzae.*	The limited resources led to competition between the species, and *C. ferrugineus* could have outcompeted *S. oryzae*.	[190]
*Lasioderma serricorne* (F.)	Wheat and wheat feed	Temperature: 30 °C RH: 60%	The presence of *C. ferrugineus* restricted the survival of *L. serricorne* in limited wheat feed. On the contrary, the presence of *L. serricorne* in wheat encouraged the growth of *C. ferrugineus.*	*L. serricorne* is a primary pest, and *C. ferrugineus* is a secondary pest, so the damage to wheat kernels by *L. serricorne* could have led to easy access to food for *C. ferrugineus.*	[190]

**Table 5:** Developed mathematical models of population dynamics and movement of *Cryptolestes ferrugineus*.

Model		Input Parameters	Highlights	References
Population dynamics	-	Development and survival rate, fecundity, and energy budget at known environmental conditions.	Predicted the number of insect life stages and bioenergetic variables similar to the observed values during population growth phase.	[206]
Potential number of generations simulation	Combination of population dynamics and heat transfer model	Initial grain/harvest temperature, storage time, and daily ambient temperature.	Data from 1952 to 1990 for each crop district in the three prairie provinces of western Canada was used. The number of generations and level of infestation mainly depend on the initial storage temperature. Using harvest temperature and date, the districts with potential outbreaks of *C. ferrugineus* could be predicted.	[207]
Spatial model	Combination of population dynamics and bin temperature model	Grain temperature, moisture content, bin diameter, grain depth, type of grain, bin wall material, latitude, hourly temperature data, wind speed, dew point temperature, barometric pressure, solar radiation, and initial insect density (number of insects immigrated into the bin).	The model was validated using field data from a bin situated in Kansas, U.S. The predicted grain temperatures were accurate at all locations except the center-top location. Due to convective air movement, the temperature at the center top portion was 8 °C higher than the predicted temperature during December.	[208]
Hot spot model	Combination of (a) a heat transfer model (three-dimensional, finite element), (b) population dynamics model, (c) heat production model, and (d) insect movement model	Grain bin properties (size, shape, emissivity), grain properties (grain depth, temperature, moisture content, specific heat, thermal conductivity, and bulk density), weather data (ambient temperature, wind velocity, and solar radiation), initial insect density, and introduction location.	Predicted the adult population, grain temperature, and location of insects, and hence the development of hot spots. Center of the grain bulk was identified as being more prone to hot spot development since it was the most suitable location for insect multiplication.	[209]
Comparison between hot spot model [209] and spatial model [208]	-	Based on Winnipeg, Canada, and Topeka, Kansas, weather data (Same input parameters as reported by Flinn et al. [208] and Mani et al. [209]).	Hot spot model included feedback from the insect model to the temperature model, whereas spatial model did not include feedback. Hot spot model is better (realistic) than spatial model in predicting the grain temperature and insect population since it considers the effects of insect movement and heat production as well as the variable heating around the bin wall.	[210]
Ecosystem model	Coupled insect distribution model with temperature model	Ambient weather data, grain bin properties, initial insect number, type of grain.	Based on weather data for Winnipeg, Canada, in 1990. Predicted that a higher percentage of adults remain at the center of the bin when high-temperature gradients and low temperatures in the boundary exist. The insect distribution mainly depends on the introduction location and the temperature distribution inside the bin.	[211]
Time-varying distributed delay model	-	Temperature, moisture content, and chronological time.	Predicted the aging rate of insects at constant or transient temperatures with various RH. Predicted the surviving rate of insects under different environmental conditions. The predicted results were compared with the experimental data from two granaries for 4 months and showed no significant difference.	[212]
Calculation of two-dimensional diffusivity	Analytical solution	Initial number of insects introduced, size of the grain chamber, movement period, and insect number in each section of a two-dimensional wheat chamber under constant environmental conditions.	Two-dimensional movement of insects follows a diffusion pattern. Diffusivity increased with increase in temperature and insect numbers and decrease in moisture content and movement period.	[183]
Population redistribution model	Modeled by transport equations and solved by finite difference method	Insect number in each section of wheat column and chamber, initial number of insects introduced, size of the grain column and chamber, and movement period.	Compared the insect redistribution in one- and two-dimensional wheat columns and chambers, respectively, and reported that there was no significant difference between the recovered numbers and those predicted by finite difference (numerical) and analytical methods.	[213]
Phenology model	Model to correlate the biological age of insects with their aging, development, and multiplication	Development rate of insect at a given environmental condition, mean lifespan of the insect at given condition with minimal stress, and chronological time.	Introduced a new term called ‘Physi-Biological time’ to normalize the distribution of insect development under different environmental conditions.	[214]

## Data Availability

Data sharing is not applicable to this article.
